# The correlations of mental health literacy with psychological aspects of general health among Iranian female students

**DOI:** 10.1186/s13033-019-0315-6

**Published:** 2019-08-27

**Authors:** Mohammad Amin Bahrami, Donya Bahrami, Kefayat Chaman-Ara

**Affiliations:** 1 Healthcare Management Department, School of Management and Medical Informatics, Shiraz University of Medical Sciences, Shiraz, Iran; 2 Health Human Resource Research Center, School of Management and Medical Informatics, Shiraz University of Medical Sciences, Shiraz, Iran; 3 Fars Education Department, Shiraz, Iran; 4 Obstetrics and Gynecology Department, Shiraz University of Medical Sciences, Shiraz, Iran

**Keywords:** Mental health literacy, Depression literacy, General health

## Abstract

**Background:**

Mental health literacy has been defined as knowledge and beliefs about mental disorders which aid their recognition, management, or prevention. This study aimed to examine the correlations of mental health literacy specific to depression and general health in a sample of high school students in Iran.

**Methods:**

A cross-sectional study was conducted during the first 6 months of 2018 among the students of an Iranian high school. A total of 65 students contributed to the study. The required data were gathered using two valid questionnaires, Goldberg and Hillier’s version of the General Health Questionnaire (GHQ-28), to measure psychological quality of life, and the Depression Literacy Questionnaire (D-Lit). The data were analysed with descriptive statistics and Pearson correlation coefficients using SPSS version 22.

**Results:**

Neither the general health total scale nor any of its subscales showed statistically significant correlations with depression-related literacy.

**Conclusion:**

Correlation of mental health literacy with general psychological health was not confirmed in this study. Also, due to the contextual differences of different societies, the generalisation of our findings should be done with caution.

## Background

Mental disorder is a behavioural or mental problem that causes significant distress or impairment of personal functioning. Mental disorders are among the most common disorders worldwide. Global socioeconomic changes in recent years, including industrialisation, rapid population growth, urbanisation, and immigration, have created a wide range of mental disorders in different societies [1]. Currently, mental illness is one of the five major diseases causing disability, accounting for more than 30% of all disabilities in a lifetime [1, 2]. In recent decades, mental disorders seem to have become a leading cause of disability and early death. According to the World Health Organisation (WHO), 500 million people around the world were reported as suffering from some kind of mental disorder in 2002 [1]. A high prevalence of mental disorders is not specific to a particular region, and all parts of the world have seen an increasing prevalence of mental disorders [1–3]. Mental disorders, in addition to causing direct disability and early mortality, also indirectly affect the incidence, progression, and prognosis of other diseases [2] and are associated with long-term disabilities [1]. It has been clearly documented that untreated mental health problems are strong determinants of poor educational achievements, as well as interpersonal, family, and social deficits and reduced life expectancy due to the associated medical conditions [4]. In short, they strongly reduce the quality of life and create a considerable economic and social burden on the individual, family, and community. For this reason, management of mental disorders is one of the main priorities of health systems in different countries today, and is an issue which has attracted considerable attention from researchers and policymakers [1, 5, 6]. Due to the high prevalence, chronic nature, and long-term negative consequences of mental disorders, immediate action including continuous monitoring of the population’s mental health and designing and implementing effective control strategies, has become a urgent need in all countries [1, 3, 7]. One such strategy is the promotion of health literacy, in particular mental health literacy, whose impact has been confirmed by many studies at the community level. WHO has defined health literacy as the cognitive and social skills that determine the motivation and ability of individuals to gain access to, understand, and make use of information in ways which promote and maintain good health. It has identified health literacy as one of the most important determinants of health status. Despite the importance of health literacy, various studies have reported widespread inadequate levels of health literacy in different societies [2, 8].

Mental Health Literacy (MHL) is a subset of health literacy first introduced by the Australian researchers Jorm and colleagues, which refers to the knowledge and beliefs about mental disorders that can aid their recognition, management, and prevention [9–11]. According to Jorm et al.’s initial definition of this concept, mental health literacy has seven components: (1) the ability to recognise specific disorders; (2) knowledge of how to seek mental health information; (3) knowledge of the risk factors of mental illness; (4) knowledge of causes of mental illness; (5) knowledge of self-treatments; (6) knowledge of professional help available; and (7) attitudes that promote recognition and appropriate help-seeking [2, 10, 12]. In general, these seven components can be classified into the three categories of recognition; knowledge of factors related to mental health; and attitudes and beliefs about mental disorders [10].

Although this definition has been widely used in research, it has come in for criticism as being problematically narrow in scope and disease-centred, as well as neglecting self-regulation techniques and strategies for improving mental health. Kusan and Wei introduced a broader definition of MHL, including knowledge to maintain and enhance good mental health, knowledge of mental disorders and their treatments, decreased stigma about mental disorders, and enhanced help-seeking efficacy [10]. Also, a more recent revision of this concept includes the ability to provide support for someone who has a mental disorder [9]. Many studies of the concept of mental health literacy have shown that adequate mental health literacy is associated with care of the disease, information-seeking behaviour, prevention of subsequent complications, and reductions in long-term harms [5, 13]. Moreover, improved health literacy improves the use of mental health services, which in turn leads to better outcomes [4, 10]. Research on influencing factors of mental health literacy has also shown that factors such as age, gender, education, history of mental disorders, and economic status are associated with the level of mental health literacy [5, 10].

However, the majority of such research has focused on the MHL status of different populations in relation to demographic characteristics. Its possible effects on the other dimensions of health, such as physical and social health, have been less studied [10]. Furthermore, MHL has been the subject of extensive study only in certain countries [14], and attention has focused on certain disorders such as depression and schizophrenia, with others such as anxiety and personality disorders being less studied [15]. Therefore, the research in this field shows gaps, especially for certain countries.

The Islamic Republic of Iran is a large multicultural country with a long history located in the Middle East. Iran is a lower-middle income country with a population of more than 80 million people according to the latest census, of whom 51% are men and 49% are women. Although population growth has declined in recent years, Iran still has the second youngest population in the Middle East and North Africa. The literacy rate in Iran is 88% and the gender equality in education has improved in the last three decades, such that today most girls attend school. According to the Ministry of Education, in the academic year of 2018–2019 about 14 million students were enrolled in Iranian schools [16, 17].

Iran spends 7% of its Gross Domestic Product (GDP) on health, and according to a WHO report, about 3% of government health expenditures in 2005 were allocated to mental health [18]. Like other countries in the world, Iran has a high prevalence of mental disorders in both the general and student populations. The first national mental survey of Iran was conducted in 1998, in which the rate of mental disorders was reported as 21% (25.9% among women and 14.9% among men). In the second National Mental Health Survey, this rate was reported to be 17.1%. However, a study in 2007 reported the prevalence rate of mental disorders in Iran as 34.2% [1]. Several individual studies have also reported a high and increasing prevalence of mental disorders in the country. Therefore, in line with global trends, Iran faces the challenge of a high prevalence of mental disorders, and estimates suggest that mental disorders rank second in Iran’s burden of disease [2]. Also, due to the young population of the country, in recent years the prevalence of mental disorders among Iranian children and adolescents has attracted great attention. A population-based survey (IRCAP) of 30,532 children and adolescents aged between 6 and 18 years across all provinces of Iran reported that 22.31% of the participants had at least one mental disorder [7]. Another National Mental Health Survey (Iran MHS 2011) reported that 21% of the participants aged 15–19 years suffer from at least one mental disorder [19]. Various other studies have also reported a high prevalence of mental disorders among students [3, 6, 20–23]. Therefore, in recent years the attention of Iranian policy makers has turned to appropriate interventions and services to improve students’ mental health.

In Iran, mental health care is provided at the three levels of primary, secondary, and tertiary care. Iran has a well-developed primary health care (PHC) system introduced in 1980 and funded by the government [19, 20]. In 1988, the Ministry of Health implemented a plan to integrate mental health care with the PHC, which has greatly contributed to the coverage of mental health, especially in rural areas and for the adult population. In addition to the health system, the Iranian State Welfare (Behzisti), the Ministry of Education, and the Special Education Organisation also participate in the provision of psychosocial care for students in Iran [19]. In recent years, the country has implemented several programs, such as students’ mental health programs, life and parenting skills training, and a mental health week in schools, which are aimed at managing mental disorders among students. Also, the Iranian Department of Education has recently begun a project, ‘Namad’, to improve the physical, psychological, and behavioural health of school students, and the Health Promoting School program has been operated since 2003 in Iranian schools [16]. In addition, the improvement of mental health literacy has been a matter of government concern in recent years. In the National Mental Health Program of Iran, mental health literacy is considered one of the 10 major issues, and its promotion in all demographic groups has been introduced as one of the three basic strategies for improving mental health [2]. However, no comprehensive study of mental health literacy and the effects of these programs has yet been published. The aim of this study was to determine the level of mental health literacy among Iranian female students and its correlation with general health. To the best of our knowledge, this study is one of the first studies in this field in the country.

## Methods

This study examined the correlation between students’ depression-related literacy and their general health through a cross-sectional method during the first 6 months of 2018 among 7th, 8th, and 9th graders of an Iranian girls’ high school [Hazrate Omolbanin (PBUH) School, Chabahar, IR Iran]. A total of 65 students participated in the study. Students who had a medical condition were excluded from the study. All participants provided informed consent to be included in the study and were assured that their personal information would be kept confidential. The parents of the students were made aware of the participation of their children in the study and had the opportunity to refuse to let their children participate in the research. The school principal and students’ teachers approved the study. Questionnaires were completed in class, and any students who were absent on the testing day had the opportunity to participate in the study the following week. All the study procedures were conducted in accordance with the ethical standards of the Declaration of Helsinki. The required data were gathered using two valid questionnaires:

1. Goldberg and Hillier’s 28-item scaled version of the general health questionnaire (GHQ-28): In this study we used the GHQ-28 to measure the psychological aspect of quality of life. The GHQ-28, developed by Goldberg and Hillier in 1978 as a screening tool to detect those likely to have or to be at risk of developing psychiatric disorders, is a 28-item instrument to measure emotional distress in medical settings. Through factor analysis, the GHQ-28 has been divided into four subscales: Somatic symptoms (items 1–7); anxiety/insomnia (items 8–14); social dysfunction (items 15–21); and severe depression (items 22–28). In our study, the participants were asked to score the items on a 4-point scale ranging from ‘not at all’ to ‘more than usual’ to ‘very much’, scored 0–3 on direct items and 3–0 on reverse answer items. The questionnaire had seven reverse items: 1, 15, 17, 18, 19, 20, and 21. The mean scores for each subscale and the total scale were calculated after the questionnaire was completed. The thresholds were assumed as in Table 1 as indicating likely to have or to be at risk of developing psychiatric disorders:

**Table 1:** The scoring threshold of GHQ-28 subscales and total scale

Disorder level	Subscales’ scores	Total score
No/very low disorder	0–6	0–22
Low disorder	7–11	23–40
Moderate disorder	12–16	41–60
Sever disorder	17–21	61–84
  The reliability of the Persian version of the GHQ-28 has been confirmed by Nazifi et al. [24], who obtained values of Cronbach’s alpha coefficient of 0.923 for the total scale and 0.865, 0.883, 0.746, and 0.897 for somatic symptoms, anxiety/insomnia, social dysfunction, and severe depression subscales, respectively.
2. The Depression Literacy Questionnaire (D-Lit) (Griffith and collegueas): the Depression Literacy Questionnaire (D-Lit), which was developed by Griffith and collegueas in 2004 to assess mental health literacy specific to depression, consists of 22 items scored true or false. In our study participants answered each item with one of two options, ‘true’ or ‘false’. Each correct response received one point, while incorrect responses received zero points. Higher scores indicate higher depression literacy. We used a standard ‘forward–backward’ procedure to translate the Depression Literacy Questionnaire (D-Lit) (Griffith and collegueas) from English into Persian. To demonstrate content validity, we used the content validity ratio to quantify the extent of experts’ agreement. The reliability of the questionnaire also was confirmed prior to the study by a value of Cronbach’s alpha of 0.70.

After completing the questionnaires, the gathered data were analysed with descriptive statistics (means and standard deviations) and Pearson correlation coefficients using SPSS version 22.

## Results

The descriptive results of the participating students’ depression-related literacy are presented in Table 2.

**Table 2:** Descriptive statistics of studied students’ depression-related literacy

No.	Item	Incorrect response		Correct response		No response		Mean	SD
		N	%	N	%	N	%		
1	People with depression often speak in a rambling and disjointed way	43	66.2	22	33.8	00	00	0.33	0.47
2	People with depression may feel guilty when they are not at fault	16	24.6	49	75.4	00	00	0.75	0.43
3	Reckless and foolhardy behavior is a common sign of depression	37	56.9	28	43.1	00	00	0.43	0.49
4	Loss of confidence and poor self-esteem may be a symptom of depression	16	24.6	48	73.8	1	1.5	0.75	0.43
5	Not stepping on cracks in the footpath may be a sign of depression	8	12.3	57	87.7	00	00	0.87	0.33
6	People with depression often hear voices that are not there	27	41.5	37	56.9	1	1.5	0.57	0.49
7	Sleeping too much or too little may be a sign of depression	28	43.1	36	55.4	1	1.5	0.56	0.50
8	Eating too much or losing interest in food may be a sign of depression	35	53.8	27	41.5	3	4.6	0.43	0.49
9	Depression does not affect your memory and concentration	22	33.8	43	66.2	00	00	0.66	0.47
10	Having several distinct personalities may be a sign of depression	20	30.8	45	69.2	00	00	0.69	0.46
11	People may move more slowly or become agitated as a result of their depression	27	41.5	38	58.5	00	00	0.58	0.49
12	Clinical psychologists can prescribe antidepressants	34	52.3	30	46.2	1	1.5	0.46	0.50
13	Moderate depression disrupts a person’s life as much as multiple sclerosis or deafness	33	50.8	31	47.7	1	1.5	0.48	0.50
14	Most people with depression need to be hospitalized	32	49.2	33	50.8	00	00	0.50	0.50
15	Many famous people have suffered from depression	42	64.6	22	33.8	1	1.5	0.34	0.47
16	Many treatments for depression are more effective than antidepressants	49	75.4	16	24.6	00	00	0.24	0.43
17	Counseling is as effective as cognitive behavioral therapy for depression	55	84.6	9	13.8	1	1.5	0.14	0.35
18	Cognitive behavioral therapy is as effective as antidepressants for mild to moderate depression	16	24.6	48	73.8	1	1.5	0.75	0.43
19	Of all the alternative and lifestyle treatments for depression, vitamins are likely to be the most helpful	28	43.1	37	56.9	00	00	0.56	0.49
20	People with depression should stop taking antidepressants as soon as they feel better	26	40.0	38	58.5	1	1.5	0.59	0.49
21	Antidepressants are addictive	35	53.8	29	44.6	1	1.5	0.45	0.50
22	Antidepressant medications usually work straight away	4	6.2	60	92.3	1	1.5	0.93	0.24
Total	Depression-related literacy	–		–		–		12.31	2.13

Table 2 shows that the students have a moderate level of depression-related literacy.

Also, the descriptive results of the students’ general health are presented in Table 3.

**Table 3:** Descriptive statistics of studied students’ GHQ-28 subscales and total scale

Item	N	Mean ± SD	Status
Somatic symptoms	62	6.98 ± 3.95	No/very low disorder
Anxiety/insomnia	62	8.11 ± 4.36	Low disorder
Social dysfunction	63	10.34 ± 3.99	Low disorder
Severe depression	65	6.04 ± 5.80	No/very low disorder
GHQ-28 total scale	57	30.21 ± 12.23	Low disorder

As shown in Table 3, the students have very low or low disorders in general health and its subscales. With regard to their general health total scores, 37.4% of the participants had low disorder and 18.3% moderate disorder.

The correlation coefficients of depression-related literacy and the GHQ-28 subscale and total scale scores are presented in Table 4.

**Table 4:** Correlation of depression literacy and GHQ-28 subscales and total scale

GHQ-28 subscales	Depression literacy	
	R	P value
Somatic symptoms	0.195	0.17
Anxiety/insomnia	0.129	0.36
Social dysfunction	0.079	0.57
Severe depression	0.051	0.71
GHQ-28 total scale	0.022	0.88

Based on the findings presented in Table 4, general health and its subscales show no statistical correlation with depression-related literacy. This finding is surprising and suggests that better literacy about depression does not affect general health.

## Discussion

The aim of our study was to investigate the correlation of MHL and general health among Iranian female students. The results of the study showed that the participants have a moderate level of MHL. There have been far fewer studies of MHL in Iran than in other countries. Noroozi et al. [5] investigated the relationship between MHL and health promoting behaviours in 378 patients in Bushehr City with an average age of 32.3 years, and reported their mean mental health literacy score as 102.75 ± 10.17 in a range of 35–160 points. Women’s mental health literacy scores were slightly higher than those of men, and strong correlations were observed between the level of education, history of mental disorders, and level of MHL. This study also found that mental health literacy is a powerful predictor and mediator of all health-promoting behaviours [5]. In another study, Karimpour Vazifekhoran et al. [25] investigated the effect of educational intervention on the improvement of MHL in patients with type 2 diabetes in Iran. This study demonstrated the effectiveness of educational interventions in improving MHL, although the authors did not provide any analysis of the MHL status of the participants [25].

Similarly, Sayarifard et al. [26] examined the knowledge of 324 Iranian students about depression. In this study, participants’ knowledge of various aspects of depression, including cognitive impairment, informed actions for seeking help and perceived barriers, beliefs about interventions, prevention, and stigmatisation, and the role of the media, were examined. This study showed that the participants’ depression literacy is weak in some respects, and appropriate educational interventions are necessary [26]. Safa et al. [27] investigated the attitude of 600 students in Khorramabad City towards mental disorders in a descriptive study, finding that 58.8% of the participants had a negative attitude and 41.2% a positive attitude toward mental disorders.

There are more studies on this subject in other countries, most of which evaluated the level of MHL among their participants. Arafat et al. [13] studied the depression literacy level of 306 1st-year students in Bangladesh. They reported the average depression literacy rate of participants as 6.55 on a 5–12 range, which reflects a poor level of literacy [13]. Arafat et al. [28] examined the status of depression literacy in 608 participants from four population groups in Bangladesh—university students, patients with depression undergoing specialised treatment, patients with chronic non-depressive physical conditions, and medical graduates not working in mental health. In this study, the literacy of participants about the symptoms and therapeutic aspects of depression was assessed as poor. In this study, 55.77% of the participants obtained less than average scores [28]. Coles et al. [29] studied the level of knowledge of depression and social anxiety in high school students at a public school in New York, finding that although participants had better knowledge about depression than social anxiety disorder, less than 50% of them had the ability to recognise depression.

Thai et al. [30] examined the depression knowledge of undergraduate students in Hanoi, Vietnam, only 32% of whom were reported to be able to correctly identify depression. Based on this finding, the authors emphasised the need for educational interventions to promote depression literacy among Vietnamese students. Such training can focus on the symptoms of depression, the intention to seek help, and first aid [30]. In a study of 99 participants from rural areas of the United States with an average age of 45.4 years, Deen et al. [12] reported that the depression literacy level of 53% of their participants was high.

In a study by Yu et al. [10] of 2377 rural inhabitants of China aged 18–60 years, 58% of the participants responded correctly to 20 items of the Mental Health Knowledge Questionnaire. Also, Ram et al. [31] investigated the level of depression literacy among health care students in South India, concluding that the level of depression literacy among the students, especially paramedical students, was weak. Similarly, Clough et al. [32] reviewed the level of mental health literacy among domestic and international students at an Australian university and concluded that the mental health literacy of domestic students was better than that of international students, but that both groups needed improvement. Mahfouz et al. [14], in a study of undergraduate students at the Jazan University of Saudi Arabia, found that more than 90% of the participants had a moderate level of mental health literacy, and in conclusion emphasised the urgent need for educational intervention [14].

Wang et al. [33] also studied the MHL status of 952 people aged 15 and above in different parts of Shanghai, reporting the correct response rates for different items of the mental health literacy questionnaire of 26–98%. Bragg et al. [34], in their research on 409 college students and 40 older adults in the US, assessed the level of participants’ MHL as poor.

In conclusion, our results and those of similar studies show that despite their geographical disparities, improvement of MHL is needed in all societies. Therefore, designing and implementing effective interventions such as appropriate educational programs seems to be necessary, as most studies, such as Sayarifard et al. [26], Noroozi et al., Bragg et al., Yu et al. and Mahfouz et al. [5, 10, 14, 34] have emphasised the urgent need for educational interventions. The effectiveness of such educational interventions has also been confirmed by Karimpour Vazifekhoran et al., Yu et al., and Perry et al. [10, 25, 35]. The roles of the educational system and the mass media are very important in this regard. Other interventions, such as the creation of community campaigns, are also helpful. Improving mental health literacy requires that improvement interventions be included in national policies [2].

Our findings about the health outcomes of MHL did not indicate a correlation between MHL and general health. The number of studies in this area is very low and a wide gap can be seen in the research on the correlation of MHL and other aspects of health. Noroozi et al. [5] found that the level of MHL is associated with all kinds of health promoting behaviours. Similarly, Yu et al. [10] reported that MHL is independently related to self-reported general health. Further research is needed to document the relationship between MHL and health status.

## Conclusions

This study examined the correlations of mental health literacy and psychological aspects of general health among Iranian female students. Although some studies have concluded that mental health literacy affects health status in a variety of ways, such an effect was not observed in our study. Therefore, further studies are needed to address this important topic. Also, the generalisation of our findings to different societies should be done with caution.

## Data Availability

The datasets used and analyzed during the current study are available from the corresponding author on reasonable request.

## Abbreviations

WHO: World Health Organization
MHL: mental health literacy
GDP: Gross Domestic Product
MHS: Mental Health Survey
PHC: primary health care
